# Understanding the User's Point of View: When the Doctor Gets Sick with Cancer and Seeks Help

**DOI:** 10.2174/0117450179241325231011070735

**Published:** 2023-10-13

**Authors:** Elena Massa, Eleonora Lai, Clelia Donisi, Mario Scartozzi, Laura Orgiano, Olga Mulas, Andrea Pretta, Giovanni Caocci, Mauro Giovanni Carta

**Affiliations:** 1 Department of Medical Sciences and Public Health, University of Cagliari, Cagliari, Italy; 2 Ematology Hematology and e CTMOHSCT Center, Businco Hospital, ARNAS “G. Brotzu”, Cagliari, Italy

**Keywords:** Cancer, Cancer treatment, Doctors, Cancer/patient relationship, Empathy, Family

## Abstract

**Background:**

When physicians confront a serious personal illness, they may discover that the transition to the “sick” role is challenging and not easy. We conducted a qualitative study in which a group of doctors with cancer (DP) was compared with a group of patients with cancer, not doctors (NDP) but with a degree of education, qualifications, and a professional role comparable to that of a doctor.

**Objectives:**

The main objective was to evaluate the effect of the diagnosis and the treatment of cancer on both the patient’s personal and professional life. It was also designed to understand the effect that the experience of cancer may have on the subsequent clinical practice of DP.

**Methods:**

The eligibility criteria included diagnosis of tumors of different sites and at any stage of disease treated with local (surgery, radiotherapy) or systemic (chemotherapy, hormonal, target) therapies or a combination of both; patients actively working. A semi-structured interview was used to collect information about the patient’s cancer experiences. In both groups, six main themes and ten subthemes were identified.

**Results:**

From July to November 2021, 59 patients were enrolled in the study. Among them, 29 were DP and 30 were NDP. The median age and gender were 55.9 years ± 9.3 SD (range 38-82 y), M/F ratio 12/17 for DP, and 56.3 years ± 8.9 SD (range 40-83 y), M/F ratio 11/19 for NDP, respectively. The main themes were: theme 1, practical aspects related to diagnosis: most of the DP did not encounter difficulties in performing the tests necessary to confirm the diagnosis of cancer, unlike what was observed in NDP. Theme 2, cancer diagnosis experience: Many DP and NDP felt prepared for their own cancer experience. Two-thirds of DP already knew their cancer prognosis from their previous background knowledge and one-third of NDP did not want to discuss the prognosis in depth with their referring oncologists for the fear of learning that their cancer had a poor prognosis. Theme 3, treatment experience: for many DP, having a professional background contributed to more active participation in care and also in the management of side effects of treatments. Most NDP were satisfied with the treatment received in the hospital and the relationship with the health professionals. Theme 4, changes in work: None of the patients from both the groups stopped working permanently or lost their job because of the disease. A higher number of DP and NDP reported a loss of interest in their job. Theme 5, changes in personal/family life and friendships: more than half of the patients in both groups developed a new perspective on their private lives. Theme 6, comfort from faith: most of the patients in both groups who followed a faith, found comfort in that faith. For DP only, we explored the theme of the change in the doctor/patient relationship. Important findings from our study included positive changes in the doctor’s clinical practice including having a more empathic relationship with patients, greater consideration of the psychological impact of cancer, and greater attention to certain symptoms of cancer reported by patients.

**Conclusion:**

This study suggests the need to know the special needs of professional patients, in particular, related to the emotional difficulties, maintenance of privacy, and the need for support on their return to work. These results can help to foster improvements in current cancer care practices.

## 1 INTRODUCTION

The risk of work-related stress in the medical field, psychological disorders, and mental health of doctors globally, are extensively discussed topics in many published scientific articles, while there is little evidence on the barriers that doctors face when they get sick with serious illnesses such as cancer. Thus, their experiences are poorly understood [1-5]. When physicians confront a serious personal illness, they may discover that the transition to the “sick” role is challenging and not easy. The doctors’ attitude to their own disease is affected by different factors: during training, doctors fall into the habit of associating the illness with patients and not themselves; during their activities, the professional status of the physician and the medical knowledge have the potential to complicate their ability to cope with difficult or terminal diagnoses [6-10]. Paradoxically, knowledge about a condition may fuel anxiety instead of alleviating the fear associated with the unknown. Interestingly, personal illness experiences can lead to novel insights about patienthood, bringing about positive changes in professional-patient subsequent clinical practice as a result of newfound empathy [5, 10, 11]. As reported by Kitzman [10], the overlapping experiences of being a healthcare worker and a patient can help create or develop strategies for improved patient care. We conducted a qualitative study in which a group of doctors with cancer were compared with a group of cancer patients, who were not doctors, but with a comparable degree of education, qualifications, and professional role. The objectives of the study were to evaluate how much and in which way the diagnosis of cancer and the treatment’s path affects personal and working life, with particular attention to the interest in work and planning both personal and professional life. Our study was also designed to understand the effect that the experience of cancer may have on subsequent clinical practice. A qualitative design was chosen to offer an in-depth understanding of the experience of an illness while locating it in its broader social context.

## 2 MATERIALS AND METHODS

### 2.1 Study Design

We conducted a qualitative observational study in which a group of doctors with cancer (doctor-patients-DP) was compared with a group of patients with cancer who were not doctors (non-doctor patients - NDP).

## 3 METHODS

### 3.1 Instrument

#### 3.1.1 Eligibility Criteria

The participants were recruited from the Medical Oncology Unit of the University Hospital of Cagliari, Italy. The eligibility criteria for both groups were: age ≥ 18 years; histologically confirmed diagnosis of tumors of different sites and at any stage of disease; patients actively working at the time of diagnosis and during therapy; patients treated for cancer with at least one of the following therapies: surgery +/- radiotherapy +/- chemotherapy +/- hormonal therapy +/- biological (or target) therapy. Written informed consent was obtained from all subjects after a full study description. For NDP, other eligibility criteria were: education level (the minimum required qualification was a bachelor's degree), job qualification, and roles (chief consultant, CEO, employee). The Exclusion Criteria were: patients who did not give informed consent to the interview; patients who had retired or had become unemployed when diagnosed with cancer. After being informed of the objectives and procedures of the study, patients who matched the eligibility criteria signed a written informed consent. All procedures were carried out under the 1964 Helsinki Declaration and its later amendments. The study was approved on March 31, 2021, by the Ethical Committee of AOU Cagliari, Sardinia, Italy, and registered with the number PG/2021/5468. A semistructured interview was used to collect information about the patient’s cancer experiences. The interviewer was a female researcher with a clinical background (medical oncologist) and with experience in cancer and healthcare research. The interviews lasted between 35 minutes on average (range: 20-55 min) and occurred in the Oncology Department, in a dedicated room, respecting everyone’s privacy and in compliance with the security regulations imposed by the COVID-19 pandemic. The interviews were not recorded, but the interviewer was authorized by the patients to write notes about their answers. The semi-structured interview was used to lead an individual informal conversation. A prolonged engagement approach was used in order to generate depth and richness in the participant’s responses. However, the patients did not have to give clear-cut answers (*e.g*.: yes/ no). The topics of the semistructured interview focused on psychological and personal life aspects, the working sphere, and the ability to plan. The same aspects were explored for both groups of patients except for the change in the doctor/patient relationship topic which specifically referred to being a doctor. At the end of the interview, and to close it, the patient was asked if he or she could describe the feelings they had experienced during the interview and to share any further stories. The open interview is reproduced below.

### 3.2 Data Analysis

**Fig. (1):**
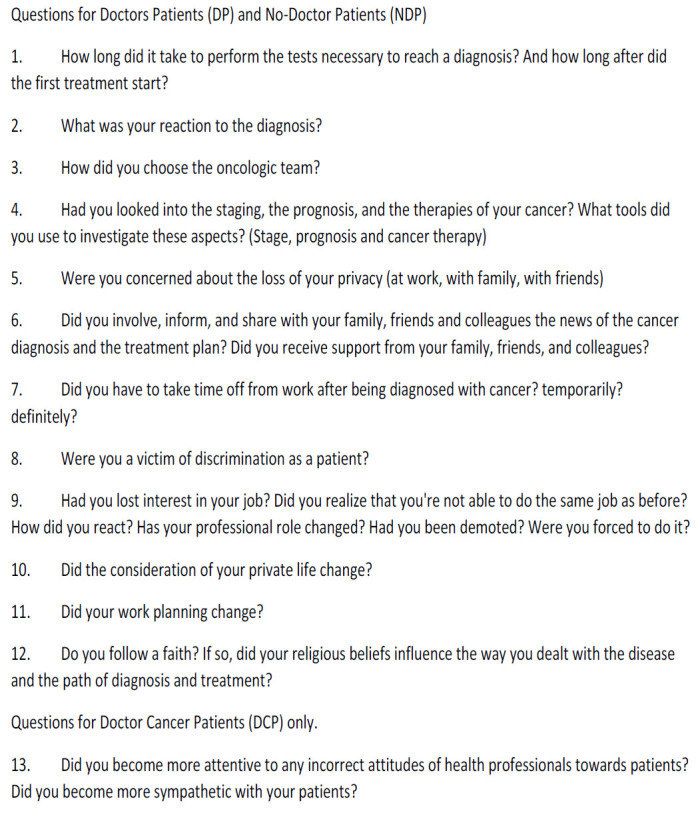
Open interview.

Thematic analysis following Braun and Clarke's five‐stage method was used [11-14]. A research team, composed of the PI (EM) and two research assistants (EL and CD) transcribed into a written form (reported in a file) with the information provided by each patient. The first phase of data analysis was to familiarize with the data by reading the file of each patient repeatedly and assessing factors that shaped the subjects' experiences. In the second phase, a preliminary coding framework was developed by two researchers (EM and CD) using a subset of transcripts, and the remaining transcripts were independently coded by EM. In the third phase, the themes and codes were checked for consistency. Discrepancies were resolved through discussion until a 100% agreement was reached. In the fourth phase, a revision of the themes was carried out, during this phase some themes collapsed into each other, and others themes were divided into separate ones. In the last phase, themes were defined and named and the data within them were analyzed (Fig. **1**).

## 4 RESULTS AND DISCUSSION

### 4.1 Sociodemographic and Clinical Characteristics of Patients

From July to November 2021, 59 patients were enrolled in the study. Among them, 29 were doctors and 30 were non-doctors. Mean age and gender were 55.9 years ± 9.3 SD (range 38-82 y), M/F ratio 12/17 for DP, and 56.3 years ± 8.9 SD (range 40-83 y), M/F ratio 11/19 for NDP, respectively. As reported in Tables **1** and **2**, the socio-demographic and clinical characteristics of the two patient groups including sex, marital status, number and age of children, educational background, job position, faith (religion or personal), stage, site of tumor and anticancer therapies are mostly overlapping. The only difference was related to the educational background; both groups of patients had university degrees, but all doctors also had specialties in addition to the degrees in medicine and surgery. The medical specialties of the doctors were the following: anesthesia, physical therapist, orthopedics, gastroenterology, endocrinology, geriatrics, oncology, general medicine, radiology, hygiene, otolaryngology, cardiology, pediatric, internal medicine, surgery, urology, gynecology. The average number of working years was 20.7 and 18.6 for DP and NDP, respectively.

### 4.2 Overview of Themes and Subthemes

In both groups, six main themes and ten subthemes were identified relating to a cancer patient’s experience. The main themes were: 1) practical aspects related to diagnosis, 2) diagnosis experience, 3) treatment experience, 4) changes in work, 5) changes in personal/family life and friendships, 6) comfort in faith. The changes in subsequent clinical practice and doctor-patient relationships were identified exclusively for the study group of doctors/patients.

The themes and subthemes explored are reported in Table **3**.

## 5 PRACTICAL ASPECTS RELATED TO DIAGNOSIS

### 5.1 Difficulties in Performing Diagnostic Tests

Most patients from both study groups immediately carried out the necessary tests to confirm the diagnosis. Only a few patients (1 DP and 3 NDP) postponed diagnostic tests by choice even though clinical symptoms of cancer were present and the suspicion of cancer was strong. Most DPS who wanted to have diagnostic tests done as quickly as possible admitted that being a doctor shortened the time to book and perform the tests needed to confirm their cancer diagnosis, while several NDPS who wanted to have tests right away, found it difficult to book an appointment and be seen within a short period. The only DP who deliberately postponed diagnostic tests reported that although it was clear to him that he needed to seek help, the busyness of his schedule and the weight of the workload acted as a barrier to seeking treatment. For a few of the NDPS, the reasons for postponing diagnostic tests were: the belief that the suspicion of a neoplastic disease was unfounded or not so strong as to have to perform the tests quickly, the fear of having the diagnosis of cancer confirmed, and the need to provide care for other sick family members as well as themselves.

### 5.2 Choice of the Oncological Team

A significant difference was observed between the two study groups in the number of specialists consulted during the diagnosis and treatment. Most of the DPS consulted only one, or at most two, specialists for the diagnosis and treatment. Many of them decided to be treated at their place of work. Having connections within the industry led to receiving a subjectively higher quality of care and facilitated the choice of the oncological team to rely on. The number of physicians consulted by NDP was much higher, with up to three specialists during the diagnostic phase and the same number in the treatment phase. The main reason for this behavior was the confusion derived from the different suggestions received from relatives, friends, and/or colleagues regarding the specialists to be consulted.

## 6 CANCER DIAGNOSIS EXPERIENCE

### 6.1 Emotional/psychological Reaction to the Cancer Diagnosis

Emotional/psychological reactions to the cancer diagnosis were very similar in the two groups, although the reasons behind the specific reactions were very different. Most of the DP and NDP reacted to the cancer diagnosis with an attitude of acceptance and determination to face the diagnostic therapeutic path as quickly as possible. Many doctors felt prepared for their own cancer experience, primarily due to knowing the various steps of the patient's path and understanding their likely prognosis. Professional patients' knowledge enabled them to recognize their diagnosis was not a death sentence. A few described a complete absence of fear. Many NDP who had already experienced cancer within their family, or through friends or colleagues, reported that they never thought they were immune to cancer. They had an idea of what they would have to face in the event of being diagnosed with cancer. Some character aspects, such as optimism and having a positive outlook on life helped these patients to face their diagnosis with determination. A small number of patients from both groups (1DP and 3 NDP) reacted to the cancer diagnosis with a severe state of anxiety that for some of them resulted in real despair. For one doctor who had cared for terminal patients, having background knowledge was not helpful; on the contrary, it led to imagine catastrophic scenarios of metastatic disease and death. For some NDP, anxiety, and desperation were due to the fear of not being able to deal with the treatment and its side effects and the fear of suffering and death.

### 6.2 Request for Information on Prognosis

Two-thirds (20/29) of DP and only one-third (10/30) of NDP did not want to discuss the prognosis in depth with their referring oncologists. Many DPS said they already know their cancer prognosis thanks to their background knowledge, while for some NDP the main reason for not asking detailed questions about the prognosis was the fear of learning that their cancer had a poor prognosis. Most of the NDP asked for information about the stage of the disease and its prognosis from the referring physician but also sought information independently *via* the Internet or from friends and family.

### 6.3 Support from Family, Friends, and Colleagues

More than half of the patients in both groups (18/29 Dps and 19/30 NDP) reported that they weren't worried about their privacy. They shared the diagnosis of cancer with family, friends, and colleagues. A subset of doctors shared the diagnosis with their family but only disclosed some information related to the disease, avoiding talking about certain topics such as stage, prognosis, survival, or mortality rate to protect loved ones from excessive worries about their health. As doctors, they knew they were the ones on whom others relied for their well-being and, at times, comfort and consolation, and they found it very difficult to admit that they had to cross over and become a patient. About a third of NDPS and a few doctors with cancer decided not to share their cancer diagnosis with colleagues in the workplace. The fear of demotion or the loss of authority in one's role was one of the main reasons for this decision. Most patients in both groups received support from family, friends, and colleagues. Only a few patients from both groups, 2 DPS, and 3 NDP, reported with disappointment that they did not receive the support, empathy, and understanding expected from some colleagues with whom they had shared the diagnosis.

### 7 TREATMENTS EXPERIENCE

### 7.1 Involvement in the Therapeutic Decision-making Process

For many DPS, having a professional background contributed to more active participation in care. Medical knowledge has enabled them to self-manage side effects and adverse events related to treatment both in the hospital and at home. Most of the doctors, despite having the possibility and knowledge to make choices about their treatment, decided to rely totally on the team of specialists chosen. Some doctors (5/29) with limited knowledge (for example, doctors whose cancer was outside of their specialty) preferred to be treated as normal patients and to receive standard information. A very small number of NDPS found that providers were reluctant to discuss the decision-making process in detail, especially regarding the use of different therapies other than the one proposed or complementary and alternative therapies.

### 7.2 Relationship with the Hospital and Health Professionals

Many DPS chose to be treated at their place of work considering an advantage, in their knowledge, and understanding of the said place. They felt it gave them the ability to move within the healthcare system that is already familiar to them. Familiarity with the context helped in the procurement of information and resources and helped to create an overwhelming environment in the hospital. During the diagnostic and therapeutic path, some DPS benefited from modification to the usual hospital practice, with some patients receiving treatment in separate rooms or in a less crowded time slot than other patients. Most of the NDPs were satisfied with the treatment they received in the hospital and the relationship with the health professionals (doctors, nurses, technicians). Few NDP (3/30) and only one DP felt discriminated against because of their illness, reporting that they felt discriminated against mainly by health professionals such as nurses or health assistants rather than by doctors. Rudeness, unkind responses, and long waits for therapies are among the behaviors displayed by the healthcare personnel that have mainly contributed to making patients of both groups feel discriminated against.

### 7.3 Treatment Tolerability

Before starting the treatment, many NDPS were afraid of the possibility of side effects, but most of them found that the treatment phase was less overwhelming than expected. Many DPS, thanks to their background knowledge, felt prepared to face the side effects of the treatments. For many patients (mainly women, but not only) of both groups, hair loss due to chemotherapy was one of the most difficult side effects to accept. Some patients of both groups suffered psychological discomfort from cancer and its treatment, including anxiety, depression, and concern for the suffering of others as a result of their disease. One of the late and longer-lasting side effects reported by patients in both groups was mild but persistent cognitive dysfunction, including a lack of mental stamina and problems with concentration and short-term memory.

### 8 CHANGES IN WORK

### 8.1 Temporary and Permanent Interruptions from Work

All patients of both groups had temporarily stopped their work for some months during the diagnostic and treatment phase. No patient had to permanently stop working or lose their job because of the disease, but two DPS and one NDP anticipated their retirement from work by a few years compared to the expected date. Significant emotional difficulties accompanied the return to work for more than a third of doctors, especially those who worked with cancer patients.

### 8.2 Loss of Interest in Work

A higher number of patients in both groups reported a loss of interest in their jobs. For the DPS, the main reasons for the loss of interest in the job were the need to take care of themselves associated with feelings of disempowerment and loss of professional identity. A handful (5/29) of DPS described finding it difficult to separate the identity of a physician from that of a patient. Some of the DPS acknowledged that they were no longer confident in carrying out their work and were afraid of making mistakes. Very few DPS (2/29) felt unmotivated to continue in their work. Despite everything, DP, after having stopped working for a few months, did not encounter any difficulties in starting to work again.

### 8.3 Changes in Professional/Work Planning

After the diagnosis of cancer, about a quarter of the DP and very few NDP changed their previous beliefs regarding their professional lives. Some DPS renounced any career advancement for various reasons such as physical difficulties (mainly fatigue), but also because the new position required an excessive mental effort, required more time away from their family, friends, hobbies, or because the new professional role also required moving to another city. A few NDP had to change some job roles due to cancer, but none of them had been demoted.

### 9 CHANGES IN PERSONAL / FAMILY LIFE AND FRIENDSHIPS

More than half of the patients of both groups developed a new perspective on their private lives. Most of the DPS after the cancer diagnosis decided to spend more time with their family (children and especially grandchildren) and friends and dedicated more time to their hobbies (music, painting, and theatre). They realized that before being diagnosed with cancer, they spent most of their time at work and that even when they were at home they often continued to work. Furthermore, some (3/29) DPS refused to spend money on a new car or a second house, whereas some NDP (3/30) decided not to spend money on trivial things. Due to the neoplasia, three female NDPs abandoned the idea of having children.

## 10 COMFORT IN FAITH

Most of the patients in both groups who followed a faith, whether it be personal or religious, found comfort in faith and spirituality at different periods of the disease.

### 10.1 Themes Identified only for the Group of Doctors with Cancer

#### 10.1.1 Changes in the Doctor/Patient Relationship

Although most DPs described themselves as empathetic towards their patients before they were diagnosed with cancer, half of them changed their approach to interacting with patients, especially cancer patients. Some of the DPs have become more careful in detecting cancer symptoms or side effects of treatments reported by patients. Asthenia, dysgeusia, sarcophobia, and mild and moderate constipation, were considered to be of minor importance because they did not endanger the patient's life. After experiencing cancer, they realized that these symptoms, although mild, have a significant impact on the patient’s quality of life. They, therefore, began to give importance to these symptoms, adopting early prophylactic and therapeutic measures to identify and counter them. Moreover, DP sometimes underestimated the symptom “pain” reported by patients, both acute pain and chronic pain. Some of them introduced the use of tools suitable for pain measurement (such as pain measurement scales) in their daily clinical practice and started to have less hesitation in prescribing drugs for moderate-severe pain, such as opioids. A subset of physicians reported to pay more attention to the patient’s psychological support, investigating any symptoms of depression/anxiety, and offering advice and support, including pharmacological ones. A non-oncologist doctor with a long personal history of cancer (25 years) from about 8 years with metastatic disease, described himself as an empathetic doctor with his patients prone to empathizing with them, even before becoming a cancer patient himself. With disappointment, he described situations in which other doctors showed no sensitivity and attention (not towards himself but towards other patients). Observing the behavior from the patient’s standpoint of another doctor towards patients enabled him to become more critical and objective in evaluating the fairness and ethics of other physicians. A young DP reported having difficulty managing patients who had received the same cancer-type diagnosis, especially if they were young females with children. She further stated that if she could, she would further avoid having to deal with these patients. The discomfort was not due to the fear of being unable to do her job well, but rather having to relive emotions, feelings, and situations that she would like to forget. One DP reported that she developed a greater ability to identify patients who suffered truly from those who only appeared to be suffering but were not suffering or had suffered to a lesser extent. Finally, DP found that as a patient, the perception of the time that doctors devote to patients in medical care was very different. The same amount of time dedicated to a patient during their activity, before they became cancer patients, was perceived differently. The time devoted to him by the doctor was perceived as shorter.

## CONCLUSION

As cancer is one of the most common chronic diseases diagnosed worldwide, it is inevitable that many working cancer health professionals will be diagnosed with cancer in their lifetime [5]. Multiple issues complicate the process of a physician assuming a new role as a patient. The present study shows that professional patients who develop a serious illness, experience unique needs, benefits and challenges which non-health-professional patients and general professional patients are unlikely to encounter. In adopting the patient role, being a doctor is an advantage in some aspects and a disadvantage in others [5, 11, 15]. The scientific knowledge of physicians, the personal and working relationships with other specialists, and the knowledge of the health system's organization allow them to solve problems related to practical aspects and to participate more actively in the management of their disease. In our study, the DP consulted a lower number of specialists, both during the diagnosis and treatment phase, compared to the NDP. We can hypothesize that this difference reflects the better self-management skills of healthcare professionals compared to the NDP. Conversely, having background knowledge made the experience of cancer fear-inducing for some DPs. Fear concerning the worst‐case scenarios of metastatic disease and death (particularly for those who had previously cared for terminally ill patients) and distress when later treating similar patients [16, 17]. Physicians who become ill face distinct privacy-related challenges. In our study, only a few DPs decided not to share their cancer diagnosis with colleagues in the workplace. The main reasons for this decision were due to the fear of demotion or the loss of authority in one's role. The main differences observed between the two groups of patients were the loss of interest in work and changes in personal/family life and friendships. One of the reasons why most of the doctors (*vs* a few NDPS) reported a temporary loss of interest in their work derives only in part from physical difficulties but more from psycho-emotional issues in dealing with clinical situations that they have experienced themselves. About a third of DPs acknowledged that they were no longer confident in carrying out their work and were afraid of making mistakes. Regarding the changes in personal/family life, friendship, and future planning, we observed a change in the doctor's approach to life after the diagnosis of cancer compared to NDP. On average, doctors work 53.3 hours per week, of which 38% report working> 60 hours per week [18-20]. We have to consider that as busy professionals with equally time-consuming family commitments, doctors tend to live like everyone else, from one activity to another, without really stopping to consider the meaning of their daily lives. Having cancer was a reason to analyze what they were doing every day, to prioritize and dedicate time to those aspects of their life that are essential. Some things that seemed important before cancer seemed trivial after a cancer diagnosis. Important findings from our study include positive changes in clinical practice, particularly in the management of cancer patients, including having more empathetic relationships with patients, greater consideration of the psychological impact of cancer, and greater attention to certain symptoms reported by patients such as asthenia, dysgeusia, sarcophobia, mild and moderate constipation. Although participants considered themselves empathic prior to their diagnosis, there was a frequently reported deepening of understanding following their diagnosis. The acquisition of self-experiential empathy has resulted in more authentic communication and more active involvement in the care of patients. Evidently, these subjective improvements in practice arose from the insights gained from having suffered from an illness that was directly related to their healthcare profession. Empathy is considered a prerequisite to having a successful physician-patient relationship, an integral part of high-quality patient-centered healthcare [21-23]. Empathetic engagement in patient care seems to exert positive influences on both patients and physicians. It has been linked with a decrease in patient symptoms like pain [24, 25] and anxiety [26], increased patient satisfaction [27-35], increased adherence to treatment [18, 23], and improved clinical outcomes [27, 35-42]. In addition, empathetic physicians demonstrate a higher level of well-being [43-45], achieve higher ratings of clinical skills [46], suffer from lower levels of burnout [45], and are at decreased risk of medical malpractice [47-49]. In our study, the DP had the opportunity to objectively observe the behavior of their colleagues not only towards themselves but also toward the other patients. Although returning to work can be difficult for some cancer survivors, for many, it represents a return to normality and aids in rebuilding personal identity [50]. Hence, DPs who bear the loss of their previous professional abilities may face further challenges in the form of a potentially complicated survivorship journey. Accordingly, these difficulties should be addressed within supportive care for DPs who are required to modify their clinical practice, or through return‐to‐work programs for those who resume their original duties. The limitations of the study concern both the methodology and the characteristics of the sample. The sample consisted of patients (both DP -e-NDP) with different stages of the disease; an early or advanced stage of cancer can affect how patients cope with any aspect of the disease. Another methodological limitation is the lack of interview recording reports. This study provides commendations from DP regarding general cancer care, healthcare professional training, and DP care. Compared to non-health professional patients, professional patients are potentially more discerning in identifying key factors contributing to high-quality care and thus represent a valuable source of “insider” knowledge [10]. Our data from DP support the frequent call in the literature for enhanced communication skills training for all cancer healthcare professionals [51-53].

## Figures and Tables

**Table 1:** Sociodemographic characteristics of Dps and NDP.

**-**	-	**Dps** **(Doctor Patients)**		**NDP** **(No Doctor Patients)**	
**Total**	-	N	%	N	%
**-**	-	29	100	30	100
**Age (Years)**	Mean ±SD (range)	55.9±9.3 (38-82)		56.3 ±8.9(40-83)	
**Gender**	Male	12	41.4	11	37
	Female	17	58.6	19	63
**Marital Status**	Married	24	82,8	25	83.3
	single/divorced/widow	5	17.2	5	16.7
**Educational Background**	Graduate	29	100	30	100
**Belief**	Catholic	20	68,9	17	57
**-**	Atheist	5	17.3	5	17
	Agnostic	4	13.8	8	26
**Job/work position**	Medical Manager / Employee	6	20.7	18	60
	Chief consultant / CEO	2	6.9	8	27
	Consultant / Private Business	21	72.4	4	13
**Actively Working at the Diagnosis**	yes	29	100	30	100
	no	0	-	0	-
**Retired (at the moment of the open interview)**	-	2	6.9	3	10

**Table 2:** Clinical characteristics of Dps and NDP.

**-**	-	**Dps** **(Doctor Patients)**		**NDP** **(No-doctor Patients)**	
**-**	-	N	%	N	%
**Total**	-	29	100	30	100
**Site of Tumor**
	Breast	10	34.5	9	30
	Lung	4	13.8	5	17
	Prostate	3	10.4	3	10
	Melanoma	2	10.4	4	13
	Colon	3	10.4	0	0
	Ovarian	2	6.9	2	6
	Thyroid	1	3.4	0	0
	Kidney	1	3,4	1	4
	Stomach	1	3,4	0	0
	Pancreas	1	3,4	0	0
	Testicular	0	0	1	4
	Uterus	0	0	3	10
	Cervical	0	0	1	4
**Stage**	I	3	10.4	3	10
	II	9	31	6	20
	III	10	34.5	9	30
	IV	7	24.1	12	40
**Systemic Therapies**	Chemotherapy + hormonotherapy	13	44.8	11	36
	Chemotherapy	3	10.3	7	23
	Immunotherapy	2	6.9	6	21
	Ormonotherapy	1	3.4	5	17
	Target Therapy	1	3.4	1	3
**Local Therapies**	Surgery + Radiotherapy	18	62.2	11	37
	Surgery	9	31	13	43
	Local-regional therapy (TARF)	1	3.4	0	0
	Surgery + Local-regional Therapy (TARF)	1	3.4	2	7
	Radiotherapy	0	0	4	13

**Table 3:** Themes and sub-themes.

**COD**	**Themes**	**COD**	**Subthemes**
**1.0**	Practical aspects related to diagnosis		-
**-**		1.1	Difficulties in performing diagnostic tests
**-**		1.2	Choice of the oncologic team
**2.0**	Cancer diagnosis experience	-	-
**-**		2.1	Emotional/psychological reaction to the cancer diagnosis
**-**		2.2	Request for information on prognosis
**-**		2.3	Support from family, friends and colleagues
**3.0**	Treatments experience	-	-
**-**		3.1	Involvement in the therapeutic decision-making process
**-**		3.2	Relationship with the hospital and health professionals
**-**		3.3	Treatment tolerability
**4.0**	Changes in work	-	-
**-**		4.1	Temporary and permanent interruptions from work
**-**		4.2	Loss of interest in work
**-**		4.3	Changes in professional/work planning.
**5.0**	Changes in personal/family life and friendships.	-	.
**6.0**	Comfort from faith		
**Themes identified only for the group of physicians with cancer**			
**1.0 **	Change in the doctor/patient relationship		

## Data Availability

The data supporting the findings of the article is available at the Department of Medical Sciences and Public Health, University of Cagliari, Italy. Corrispondence to Elena Massa (dinetto13112012@gmail.com), as approved by the Ethical Committee of Sardinian Region, Italy (reference number PG/2021/5468).

## STANDARDS OF REPORTING

COREQ guidelines were followed.
